# Exploring Indigenous Ways of Coping After a Wildfire Disaster in Northern Alberta, Canada

**DOI:** 10.1177/10497323211009194

**Published:** 2021-05-11

**Authors:** Stephanie Montesanti, Kayla Fitzpatrick, Tara Azimi, Tara McGee, Bryan Fayant, Lorraine Albert

**Affiliations:** 1 School of Public Health, University of Alberta, Edmonton, Alberta, Canada; 2 Department of Earth and Atmospheric Sciences, Faculty of Science, University of Alberta, Edmonton, Alberta, Canada; 3 McMurray Métis Local 1935, Fort McMurray, Alberta, Canada; 4 Mikisew Cree First Nation, Fort McMurray, Alberta, Canada

**Keywords:** Indigenous communities, disaster, wildfire, coping, strength, Canada, northern Alberta, community-based participatory research (CBPR)

## Abstract

In May 2016, a wildfire devastated a northern region of Alberta, Canada, resulting in negative consequences on physical and mental stress, social relationships, and overall resilience among Indigenous residents. Research on coping and managing stress following a disaster has failed to incorporate unique characteristics from Indigenous perspectives. Sharing circles were held in urban and rural community settings to capture: (a) Indigenous perspectives of coping, (b) individual and collective strengths that helped Indigenous residents and communities to cope during and after the wildfire, and (c) intergenerational experiences of coping from stress among Indigenous residents. Indigenous residents’ experience with coping from the wildfire was shaped by: (a) heightened physical and emotional stress, (b) existing structural inequities, and (c) strong community cohesion and connection to culture. An unexpected outcome of this research was the therapeutic value of the sharing circles for participants to share their experience.

## Background

On May 1, 2016, a wildfire swept through the Regional Municipality of Wood Buffalo (RMWB) in northern Alberta, Canada, causing a mandatory evacuation of 88,000 residents and the loss of 2,400 homes and commercial buildings. The wildfire (also known as the Horse River wildfire) was the costliest natural disaster in Canadian history, with an estimated cost of US$8.9 billion (KPMG International, 2017), and resulting in community devastation, destruction, loss of homes, job insecurity, financial loss, injuries, impacts on mental health, and displacement and separation from loved ones. Disasters are examples of stressful situations that may challenge not only the individual but also communities’ ability to cope with what can be unexpected and sudden stress.

Indigenous residents in the RMWB region were deeply affected by the Horse River wildfire. Many Indigenous communities do not have the necessary resources, capacities, and infrastructure to adequately mitigate, respond to and recover from disaster events (Beverly & Bothwell, 2011; Finlay et al., 2012). Preexisting social and economic inequities and past historical trauma also attribute to Indigenous residents’ experience of a disaster evacuation and displacement (Dodd et al., 2018; Dunbar-Ortiz, 2014). For instance, Indigenous communities often experience poorer access to health care and significantly higher rates of disease, which further exacerbates vulnerabilities and their ability to cope during a disaster (Asfaw et al., 2019, 2020). Furthermore, past experiences have shown that the impacts of disasters can have severe consequences for the health, livelihood, and the economic well-being of Indigenous communities (Christianson, 2015; McGee et al., 2019). There is ample evidence that if a disaster occurs, evacuations and long-term displacement will have negative consequences on physical and mental health, social relationships, and overall resilience (Montesanti et al., 2019).

However, disasters and emergencies have also been found to result in some positive coping experiences, including strengthened relationships and social networks. The role of social capital and community cohesion, for instance, can enable communities to cope with emergencies under postdisaster conditions (Asfaw et al., 2019; Bihari & Ryan, 2012; Langer & McGee, 2017). During a disaster event, social cohesion is evident through the willingness of residents to help one another out during the rebuilding of their community (Carroll et al., 2005).

Contemporary definitions of coping generally include personal, family, and community characteristics that contribute to individuals’ abilities to heal in the face of adversity and manage stress (Lazarus & Folkman, 1984). However, these definitions are derived largely from research involving non-Indigenous populations and may fail to incorporate unique characteristics from Indigenous perspectives. To address this knowledge gap, our research sought to explore Indigenous peoples’ perceptions and experience of coping in the aftermath of the Horse River wildfire. The objectives of this research were to capture (a) Indigenous perspectives of coping, (b) individual and collective strengths that helped Indigenous residents and communities to cope during and after the wildfire, and (c) intergenerational experiences of coping from stress among Indigenous residents.

## Study Area and Context

The region is home to five First Nation communities, including Mikisew Cree First Nation, Athabasca Chipewyan First Nation, Fort McKay First Nation, Fort McMurray First Nation, and Chipewyan Prairie Dene First Nation. This region is also home to five local Métis organizations located in urban and rural communities across the region. Like other residents of the RMWB, many Indigenous people and communities evacuated due to the fire or smoke and spent several weeks away from their homes, jobs, schools, and communities. However, when it was time to return, many of Fort McMurray’s urban Indigenous residents did not have houses to return to. Outside the Fort McMurray urban center, nearby Indigenous communities were also affected by the wildfire when thousands of evacuees sought refuge in their communities during the initial evacuation. For Indigenous peoples and communities that did not evacuate during the wildfire, the almost complete “shut down” of Fort McMurray made accessing food, medical services, employment, and other resources difficult for the duration of the evacuation.

## Literature Review

### Stress and Coping Strategies

Coping refers to “cognitive and behavioral efforts to master, reduce, or tolerate the internal and/or external demands that are created by the stressful transaction” (Folkman, 1984, p. 843; Lazarus & Folkman, 1984). People use a variety of strategies to cope with stressful events. One strategy is the regulation of emotions or distresses that result from a stressful situation (*emotion-focused coping*). The other is the management of the problem that is causing the stress (*problem-focused coping*). Although both forms of coping are used in most stressful encounters, they are dependent of the way one appraises the stressful situation (i.e., as a threat, harm, loss, worry about a loved one, and/or a challenge; Aldwin et al., 1996). Both forms of coping rely on a range of promotive and protective factors that support resilience on multiple levels, including individual, family, community, and society. These factors influencing coping abilities can be conceptualized as individual assets (e.g., emotion regulation skills, problem-solving skills, planning, and decision-making skills, reflectiveness and personal awareness, faith, spirituality, and humor) and contextual and environmental resources (e.g., emotional security, family cohesion, connection to culture, positive social support networks, strong cultural identity, and community well-being and cohesiveness) (Jogen et al., 2020).

### Sociocultural Understandings of Coping

More recently, an understanding of coping has been expanded to include social and cultural coping resources (Chun et al., 2006; Taylor et al., 2007; Wong & Wong, 2006). Scholars have argued that coping must be viewed as a social and collective process (Chun et al., 2006; Taylor et al., 2007; Wong & Wong, 2006). Aldwin (2007) postulated a sociocultural conceptualization of stress-coping that emphasizes the social context of the stress and coping process. According to Aldwin, social context is deeply embedded in one’s cultural context. This framework views coping as a function of an individual’s social and cultural environment. Under this perspective, individuals’ experiences with cultural expectations and resources affect their perception of the demands of a stressor and of their available resources to manage or regulate the stress. This in turn affects how they appraise the stressful encounter. Moreover, research on culture and coping have begun to identify cultural variability in coping preferences and patterns across racial and ethnic groups (Gaylord-Harden & Cunningham, 2009; Joseph & Kuo, 2009; Lam & Zane, 2004), culture (Rivera, 2012; Torsch & Ma, 2000), gender (Enarson & Chakrabarti, 2009; Ginige et al., 2009; Lawrence et al., 2006; Matud, 2004; Renk & Creasey, 2003), and across intersecting categories of age, gender and race (Sheu & Sedlacek, 2004; Spence et al., 2007).

Moreover, scholars have highlighted the importance of ‘‘collective coping’’ in communities (Constantine et al., 2002, 2005). The notion of collective agency and related constructions such as feelings of solidarity, a sense of community, and social capital are all resources for collective coping that have been cited in the community resilience literature (Townshend et al., 2015). Social capital as a resource for coping is expressed through a person’s relationships within their social networks (Chan et al., 2006). Moreover, local knowledge also represents another means of accessing shared coping resources. Societies and cultures share an interdependent relationship with their environment. This relationship produces cultural interpretations and practices that allow societies and cultures to manage and cope with natural disasters (Heidi, 2005). Studies of disaster effects in Indigenous communities have focused on local understandings of disaster and coping strategies that are critical for the development of safer and sustainable communities (Heidi, 2005) and to advancing the physical, mental, emotional, and spiritual health and well-being of Indigenous peoples (Thomas et al., 2015).

### Indigenous Coping and Resilience

There is a paucity of research that has examined Indigenous perspectives and experiences of coping during a traumatic and stressful event, such as a wildfire. Research on Indigenous resilience can be useful in understanding (a) the political, social, economic, and environmental realities of Indigenous communities and (b) the range of protective and strength-based factors that support healing and recovery for Indigenous peoples from a disaster event. Albeit diverse based on various unique histories, cultures, and languages, Indigenous perspectives of healing and recovery are often grounded at a cultural level and are focused on community relationships, identity, connection to land and culture across generations (Asfaw et al., 2020; Kading et al., 2019). For example, a qualitative study by Kading and colleagues (2019) explained that Anishinaabe healing included a sense of identity, community connectedness, traditional knowledge, responsibility to family, respect toward others, ethnic pride, and connectedness with land (Kading et al., 2019). Indeed, notions of healing here illustrate a strong link with community, social connectedness, and culture and are largely based on processes of resistance to a history of oppressive colonial systems, discrimination, and loss. Therefore, further research on Indigenous peoples’ coping strategies and experiences is needed to gain a better understanding of coping processes among Indigenous peoples following a disaster.

## Method

Our study followed a community-based participatory research (CBPR) approach (Fletcher, 2003) to support engagement with Indigenous Elders, adults, and youth residing in urban and rural communities within the RMWB. CBPR results in active and informed participation by communities to establish directions of the research (Rice & Ezzy, 1999). Research in each community setting was conducted in partnership with a Community Advisory Committee and community-based Indigenous researchers. The Community Advisory Committee was developed with guidance from the local Friendship Center and the local Métis organization in urban Fort McMurray, who were partners on this study, and included six advisory members. The committee comprised Indigenous and non-Indigenous health service providers, as well as representation of Indigenous Elders from First Nation and Métis ancestry, including Dene-speaking and Cree-speaking members. The research team engaged the Community Advisory Committee in all phases of the research, valuing and legitimizing their knowledge and balancing power relationships among community and researchers throughout the study (Baum et al., 2006; Kendall et al., 2011). Historically, research with Indigenous peoples in Canada has perpetuated injustices and has had damaging effects on communities (Fletcher, 2003). Attending to power imbalances in research collaborations is a key component of CBPR, and necessary for establishing respect and trust between the researchers, community advisors, and those being researched (Baum et al., 2006; Fletcher, 2003). In addition to following the principles of CBPR approach, the research team also followed the OCAP™ principles to guide reciprocal and relevant research. The OCAP™ principles are *ownership* of cultural knowledge/data/information by the community or group; the right of Indigenous peoples to seek *control* of all aspects of research and information management processes that affect them, the right of Indigenous peoples to *access* information and data about themselves and their communities, and to manage access to their collective information; and the right of Indigenous peoples to physically *possess* the data collected (The First Nations Information Governance Centre, 2014).

The following questions were used to guide conversations in our sharing circles:

1. What are some individual- and community-level strengths that helped you during and after the wildfire?
2. What does it mean to you to cope from a traumatic or stressful event like the wildfire?

Sharing circles are a tradition for many Indigenous peoples in North America and were used as an Indigenous methodological approach in this research to capture intergenerational perspectives and experiences of coping from Indigenous residents, during and after the wildfire. Traditional sharing circles vary across Indigenous cultures and are described generally to represent important principles in the Indigenous worldview and belief systems, namely, interconnectedness, inclusiveness, continuity, and nonjudgmental active listening (Lavallée, 2009; Loppie, 2007). In sharing or talking circles, participants share all aspects of the individual—heart, mind, body, and spirit—and permission is given to the researcher to report on the discussions (Bohanon, 2006; Lavallée, 2009; Loppie, 2007). The *circle* represents unity, reciprocity, and inclusivity. Indigenous peoples enjoy a strong affinity with the circle because it symbolizes and resembles many cycles in the natural world, such as the sun and the moon, and the wind as it whirls in circles. In other Indigenous communities, it is also a symbol of equity, where community members come together to share and learn from one another in a safe space (Carr et al., 2020; Latimer et al., 2018). The use of storytelling also incorporates decolonizing methods that break down the power hierarchy between researcher and participant and enables both parties to engage in storytelling (Loppie, 2007). This approach is inherently relational and aligns with an Indigenous worldview that honors storytelling as a means of transmitting knowledge and lived experience (Tachine et al., 2016).

Sharing circles incorporate qualitative research techniques similar to group discussions and focus groups (Loppie, 2007). Similar to other qualitative research methods, sharing circles will allow the participants to steer the conversation and discuss topics they consider important and relevant. In addition, as storytelling or narrative is an essential component of many Indigenous cultures, sharing circles will allow community members to share their experiences and views in a format aligned with their culture (Loppie, 2007). How they differ from focus groups is the sacred meaning they have and by incorporating a culturally appropriate approach that allows for Indigenous protocols to be included (Carr et al., 2020; Lavallée, 2009). Furthermore, with focus groups, participants tend to limit personal information and experiences whereas sharing circles encourage storytelling, allowing participants to make connections to past and future generations of family, community, and nation (Archibald, 2008; Hopkins, 2007). There are various purposes for sharing circles that can include sharing as a means of coming together for strengthening social and community cohesion, to heal from trauma, or to learn from the sharing of local knowledge and wisdom (“Mi’Kmaw Spirituality—Talking Circles,” 2016). Our research study utilized sharing circles to answer the research question:

**Research Question 1:** How did Indigenous people residing in the RMWB cope during and following the 2016 Alberta wildfire disaster?

### Participant Recruitment and Data Collection

Participants were recruited using purposive sampling through the research team’s preexisting relationships with community leaders, local service providers, and with the assistance of our Community Advisory Committee. This recruitment method allowed for a representative sample of diverse Indigenous residents across the region. A maximum variation sampling strategy was used to guide the selection and recruitment of participants across First Nation and Métis ancestry, and from urban and rural communities. We began with sharing circles in urban Fort McMurray at the local Friendship Center. Flyers inviting residents to attend the sharing circles were posted at the Friendship Center, local Métis offices, and community health centers. Word of mouth was also used to inform community members of the sharing circles. For the inclusion criteria participants had to be living in RMWB at the time of the wildfire, be Indigenous, and fall within any of three age groups: youth (16–25 years old), adults (26–60 years old), Elders (61 years old or specified as an Elder by community). The age categories were selected by the Community Advisory Committee. We held three concurrent sharing circles at the local Friendship Center in urban Fort McMurray consisting of an Elders (*n* = 7), adults (*n* = 4), and youth (*n* = 5) circle, and occurring 1-year post-wildfire. We observed low attendance from rural Indigenous residents at the urban sharing circles, which led the research team to organize a fourth sharing circle in a rural community outside of Fort McMurray that would be accessible for rural residents. This rural sharing circle was held at a local community center 2 years post-wildfire with both adults (*n* = 3) and Elders (*n* = 9). As youth from the rural communities tend to reside in the urban Fort McMurray while attending school, they did not participate in the rural sharing circle. However, several youth residents from the rural communities outside Fort McMurray participated at the urban sharing circle described above. The delay in conducting the fourth sharing circle was due to the time required to establish trust and relationships with the community prior to conducting the sharing circle. Community research assistants spent time in the community on a regular basis to build relationships and develop an understanding of the local context. Thus, establishing strong community relationships was an important component driving our research process.

Sharing circle facilitators included a researcher and a community-based research assistant dyad. Facilitators were given a guide to facilitate the sharing circle that they were assigned, which included background information on the research study, procedures for facilitating a sharing circle, and guiding questions for participants. The facilitators explained the purpose of the sharing circles and requested and obtained verbal and written consent to audio record the conversation. Participants were encouraged to speak in their own language if they wished and a translator was available to support the team with transcription. Sharing circles began with a smudging ceremony and prayer. Smudging is an important traditional ceremony in the territory that involves prayer and burning of dried plants, such as sage or sweetgrass (McCampbell, 2011). In total, we engaged 29 diverse Indigenous residents from the RMWB for our research study. All participants in the sharing circles received gift cards for their participation.

#### Ethical considerations

The study was reviewed for its adherence to ethical guidelines and approved by a Research Ethics Board at the University of Alberta (REB Pro00070845). All participants in the sharing circles were informed and fully consented to participate in the study. Participants were assured that they could withdraw from the study up until data were analyzed without any consequences. All personal details were kept confidential and secure.

#### Data analysis

Audio recordings for all four sharing circles were transcribed verbatim. Two researchers listened to the audio files and read the transcripts line by line to generate a set of preliminary themes using the qualitative software program QSR NVivo (Version 11). In addition, researchers reviewed memos and facilitator notes from a postsharing circle debrief meeting with facilitators. The principal investigator (Stephanie Montesanti), team researchers (Kayla Fitzpatrick, Tara Azimi), and Indigenous community partners (Bryan Fayant, Lorraine Albert) developed the initial set of preliminary themes. We relied on a qualitative descriptive analytical approach, which is dynamic and reflexive (Kim et al., 2017). Qualitative description lies within the naturalistic approach to qualitative data analysis, which creates an understanding of a phenomenon through accessing the meanings participants ascribe to them (Sandelowski, 2000). Prior research explains that the goal of qualitative description is to describe experiences related to a particular phenomenon using participants’ language, whereas grounded theory, phenomenological, and ethnographical research aim to produce a theory (Kim et al., 2017). With this, the researcher works hard to stay close to the surface of the data and events (Sandelowski, 2000) where the experience is described from the viewpoint of the participants (Sullivan-Bolyai et al., 2005). Furthermore, a qualitative descriptive approach agrees with the procedures we implemented, namely, purposive sampling and the use of semi-structured interview guides (Neergaard et al., 2009; Sandelowski, 2000). Aligning with qualitative descriptive approach, content analysis was the primary strategy for data analysis (Neergaard et al., 2009; Sandelowski, 2000). Data were analyzed using codes and categorizes to determine patterns of words and phrases used (Gbrich, 2007; Pope et al., 2006).

Following data analysis, preliminary findings from the sharing circles were shared with participants who then helped to further develop the themes. The final iteration of themes was reviewed and approved by our two Indigenous community research partners (Bryan Fayant and Lorraine Albert) who are co-authors of this article. This is a key step in the OCAP™ principles (The First Nations Information Governance Centre, 2014) and also provides member checking to reduce researcher bias in data analysis (Birt et al., 2016). To further enhance the rigor of our analysis, the researchers followed a four-step framework by Whittemore et al. (2001): (a) attention to the voices of participants (authenticity), (b) reflect on how believable the results are (credibility), (c) critically appraise all decisions made during the research process (criticality), and (d) researcher to demonstrate ongoing reflection and self-criticality (integrity).

## Results

When we asked participants to talk about individual and community strengths that helped them cope or get by during and after the wildfire, they began by sharing how they were affected by the wildfire—personally, within their family unit, or community. Participants conveyed that it was important for the research team to understand Indigenous residents’ experience with the wildfire and in a “postdisaster landscape,” where the legacies of colonialism have contributed to communities often being situated in more vulnerable, disaster-prone areas. Three main themes emerged from our sharing circle conversations with Indigenous Elders, adults, and youth across the region that highlighted personal and community-level determinants of coping among Indigenous residents during and after the wildfire. How well Indigenous residents coped was shaped by the following experiences: (a) heightened physical and emotional stress, (b) existing structural inequities, and (c) strong community cohesion and connection to culture. We captured intergenerational stories and perspectives within each theme described below.

### Heightened Physical and Emotional Stress

Participants from all three sharing circles reflected on personal experiences with the wildfire that brought about heightened stress and anxiety for many communities, and ultimately affected how they coped from this event. The impacts on health and well-being among Indigenous communities were described throughout sharing circles and include impacts on physical, mental, emotional, and spiritual well-being. Some of the perceived impacts on physical health included respiratory issues and challenges with accessing necessary medications, primary health care, and speciality care services. One male Elder experienced poor respiratory health after the wildfire and described the presence of dense smoke during the summer months that resulted in significant health risks for residents:

*. . . since the fire I have had a lot of health problems, including troubles with breathing. I have never been sick in my life. I hadn’t seen a hospital or doctor for years, but now, since the fire, I have to go every Tuesday to see the doctor. Maybe because the pollution or the smoke, but I think the fire is the reason [for my poor health]*.

Participants in the adult sharing circle mainly included parents of young children or teenagers who experienced multiple disruptions in their personal lives due to the wildfire such as, the closure of schools, separation from family and friends, or loss of their home. Participants in this sharing circle also raised their concern for the health and mental health of children and elderly parents who are particularly vulnerable during natural disasters.

Alongside residents’ concerns about their health and well-being, participants also described the help-seeking barriers they encountered when accessing health care services during the evacuation. For instance, one Elder was no longer able to continue his cancer care treatments during the evacuation period. Upon returning home he experienced long delays in resuming his medical appointments and treatment. In addition, a mother in the adult sharing circle described feeling neglected during the evacuation when she attempted to seek support for her son who has complex health issues. She explains:

*. . . my son . . . has epilepsy and he’s got ADHD so we can’t just try and keep him on a cot [in the evacuation centre in Edmonton] . . . and I was expected to get proof of his medication history . . . just for them to put us up in a hotel . . . they’d [put us up] in a hotel but they wouldn’t pay for us to get there, we had to figure out our own way to get there*.

Participants also described forgetting their health cards at home when they evacuated which made it difficult to access varied health services, including refilling pharmaceutical prescriptions.

In addition to the physical health concerns experienced by residents and the challenges they encountered with the health care system during the evacuation, participants described impacts on mental, emotional, and spiritual well-being stating elevated levels of stress, isolation, and loss. All participants conveyed the indirect impacts that the wildfire had on their emotional well-being. Loss of land, cultural and personal possessions, as well as community feelings of isolation resulted in significant distress among residents. Elders were distraught when sharing their experience with displacement, destruction of the land and disruption of traditional land-based activities. A male Elder described:

*Nature is all gone now, I used to see deer and rabbits on the road, but now after that fire there is hardly any traps for hunting. You travel towards Saskatchewan and there are no traps, there is hardly anything, no wildlife.*

Elders, adults, and youth all expressed feelings of loss, whether it was a loss of their homes that burnt down or loss of sentimental and cultural belongings such as fiddles, guitars, drums, and family portraits. Elders who were dependent on hunting and foraging expressed feeling a sense of loss and uncertainty about the future viability of these activities and the related consequences for their food security and well-being. One Elder was saddened by the loss of her grandmothers’ traditional ceremonial possessions. Participants shared that many of their lifelong friends and relatives who lost their homes in the wildfire did not return back to the community. For them, this meant a loss of their social support network. Coping with these rapid life changes was difficult for all participants and this is expressed by one participant who shares:

. . . *it still gets really emotional, you raise your kids here, you have your neighbours that have been here all your life, [and] it’s all gone*. (Female adult)

Youth who evacuated to Edmonton also described feeling socially isolated and were unable to reach family members and friends during separation. As described by one male youth, “I was hoping for wi-fi [in the evacuation centre] . . . I just wanted to stay in contact and reconnect with my family and friends.” Another female youth participant added, “We had to split up, that was really hard on me, having my family all split up . . . .” Furthermore, youth also felt disconnected from their normal day-to-day activities. They went on to explain that there were no programs and activities offered to children and youth in evacuation centers or hotels.

Moreover, participants described feeling alone and not knowing where to go to for support. A female Elder explained,

*I was all by myself, nobody cared for me I just looked after myself, and nobody questioned where I was and I didn’t know where to go. I heard someone say something about hotels that were taking evacuees but there were none*. (Female Elder)

Uncertainty and fear were common emotions expressed by participants, and this is reflected in the following quote by one participant:

*. . . I ended up with my elderly mom in the vehicle and I said mom I think we’re going to die so let’s go and pray before we die. So [we] went and held hands by a tree and we prayed . . .* (Female adult)

Compounding by health stressors, participants described many structural inequalities that were a hindrance to the well-being of Indigenous communities during the wildfire as further elaborated in the next theme.

### Structural Inequities

Structural inequities include institutional and systemic drivers (e.g., racism, discrimination) that contribute to the unfair distribution of services. A crucial component of this are the policies that perpetuate structural inequities in communities. Structural inequities in the context of this research relate to how the evacuation, response, and recovery were managed by politicians and external organizations (Baciu et al., 2017). Disorganization with the evacuation and recovery response led by municipal, provincial and federal governments and poor communication from policy leaders were felt to contribute to participants’ stress and concern about their safety. Therefore, this had significant impacts on how well residents coped from the aftermath of the wildfire. A male youth participant described the evacuation of Fort McMurray to resemble a “zombie movie” as he recalled evacuees driving their vehicles off the road to bypass other drivers who were fleeing Fort McMurray. Elders spoke about the limited evacuation information shared with communities—for example, where to evacuate to, and supports available to evacuees.

First Nation and Métis communities located on the outskirts of urban Fort McMurray hosted evacuees from the city during the initial evacuation, until they were later advised to also evacuate due to the spread of fire and smoke. Participants described how this added additional stress on rural communities in particular who were already grappling with limited resources. For instance, communities that hosted Fort McMurray evacuees experienced a shortage of food, gas, and basic supplies:

*. . . we opened up our community centre, he [the chief] flew food into [our community] to make sure there was food, people were emptying out cupboards at home. They did everything possible to help with the evacuation.* (Male adult)

Elders from rural First Nation communities in the region pointed out the limited support their community received from the municipality during and postevacuation, in comparison with the institutional support provided to Fort McMurray evacuees. One male Elder conveyed,

*When we returned home, we needed help to restock our own fridges or to help our people [and] there was no services to help us*.

Another female Elder shared an example of how supports and resources were distributed unequally across rural and urban communities, as well as among residents within the same community. This experience is illustrated in the following quote:

*The teachers here in the community were compensated with new mattress’ and fridges because their food went bad. None of our community members got compensated. The teachers only live a few kilometres from me. It is sad to say but they are white people and we are native, they got compensated and we didn’t.*

Other participants shared the challenges they faced when it came to rebuilding their homes due to the cost of construction, permit requirements, and lack of insurance coverage. A male Elder participant stated,

*. . . what I’m seeing in our community is probably about 50 percent of particularly the native people are not going to be able to come back into the community that they live in, grew up in and were a part of because of the economic cost of rebuilding.*

Some Elders expressed frustration when dealing with insurance companies and the length of time it took to hear back on the status of their rebuild. In some instances, participants explained that their insurance was outdated and/or they were unaware of their insurance policies:

. . . *the insurance guy told me your lucky your home didn’t burn because you didn’t have enough insurance. I would have been homeless.* (Female Elder)

Many participants felt they had little to no support to help them navigate the rebuild of their homes, permit requirements, and insurance coverage. Although this disaster highlighted the structural inequities for Indigenous peoples in RMWB, a major strength noted in this population was the generosity and ability to come together as a community and connect to culture, spirituality, and tradition.

### Strong Community Cohesion and Connection to Culture

While disasters can change community relationships and cohesion resulting from the separation of families and loved ones, participants described how the fire “brought everyone together.” Participants reflected on their connection to community and kinship that helped them to cope and stay strong throughout the disaster. For Indigenous peoples and communities, connectedness to family, their nation, culture and spirituality are important to their identity. Elders, adults, and youth participants all spoke about how they bonded with other community members through shared experiences with evacuation or healing together through ceremony.

A female participant in the adult sharing circle described how during the crisis she noticed people felt connected and comfort in knowing that they are not alone. She explained,

*. . . everyone wants closeness, a sense of belonging. . . there is a comfort even if it means just standing next to somebody and being good company.*

Further contributing to feelings of connectedness were stories of generosity and opportunities to participate in cultural activities in other host communities. As one Elder described,

*We went to Saddle Lake to our friend’s house. They’re really traditional native people, Cree people, and they do ceremonies and sweats and stuff like that, so we felt good going to that home. And they just took us in no problem . . . good to see you, like it was nothing. We would have done the same thing if that happened to them.* (Female Elder)

While family and friends offered support to evacuees, there were also stories of how the wildfire brought together individuals who were not previously connected. One female adult recalled a memory from when she was in a donation center:

*The fires made everyone equal, Indigenous or non-Indigenous, we were all picking out Haines underwear from the same bin.*

This participant illustrated how all evacuees regardless of race, culture, or beliefs connected in some way because of their shared evacuation experiences, whether being relocated or having lost a home.

Some participants connected with one another and their community through spirituality and faith to stay strong after the wildfire by participating in cultural and traditional practices, which promoted mental and emotional health and well-being. Residents made sense of the tragedies using prayer, teachings, and faith, as noted by one male Elder “the thing that made us stronger were prayers.” A male participant in the adult sharing circle spoke about her connection to her faith and how this helped her to cope:

*I am just going to do the best I can and I am not going down, so I just am on my own feet and God is doing the heavy lifting.*

Some Elders spoke about their Indigeneity as strength making reference to the challenges Indigenous peoples have historically faced in Canada and their ability to cope through difficult times, this is underscored in the quote below:

*I think native people have an ability to cope with things because they generally had a hard life. So many things that happened in our life, it’s very . . . growing up as a native person in Canada, you have to learn to how to be strong. I think it’s just innate in us to cope. My parents . . . from the trapline, they experienced any kind of hardship you can imagine, and I think it trickles down to their kids, you know.* (Male Elder).

Another female Elder described that the wildfire was not the most traumatic event they had experienced, rather they recalled previous traumas in their life and talked about the fire as “just another thing to have to deal with.” Furthermore, a male youth participant described how he drew strength from his spirituality and the teachings from his Elders:

*. . . my grandpa taught me that whatever you build in this life you don’t take it to the next, the only thing you take is your spirituality . . . the things you gain emotionally and spiritually you take with you . . .*

The opportunity to share their story about their wildfire experience was described by participants to be an important step toward coping and healing. One prominent discussion that was exposed throughout the sharing circles, centered around mental health and the wildfire. This experience was shared by one male participant in the adult circle:

*Yeah, it really makes you sit in front of the face of whatever it is that’s bothering you, . . . getting in a meditative state where you can let go of things, but you can only let go once you’ve acknowledged the problem . . . That decompression really helped me.*

Some participants shared their appreciation for the sharing circles hosted by our research team. The sharing circles took place 1 year after the wildfire and for some participants; this was the first opportunity where they shared their disaster story. One participant explained,

*. . . it’s been a really painful thing to talk about our experiences, and I don’t know how we ever managed to get through it . . . [there are] so many things I could talk about all night, so it’s good to be here.* (Female adult)

Therefore, sharing personal stories of the wildfire allowed participants to connect over similar experiences of stress, loss and fear. While the wildfire resulted in impacts on health and well-being, and reinforced structural inequities, Indigenous residents relied on their relationship to their community and culture as a coping resource.

## Discussion

This research study sought to understand Indigenous coping experiences during and after the Horse River wildfire in northern Alberta. Prior to sharing stories of coping with the stress from the disaster, Indigenous participants felt it was important to first contextualize their experience, by sharing their lived realities of the wildfire evacuation and recovery with the research team. Our research identified several factors that affected the coping responses of Indigenous residents and communities during and after the wildfire, as well as the ways in which they coped during this difficult and traumatic experience.

At the individual level, chronic stress and health concerns resulting from the wildfire were acknowledged as a key component that affected the well-being of Indigenous residents and their ability to cope. Displacement and separation from loved ones significantly affected residents. There is extensive research demonstrating that evacuations and long-term displacement have negative consequences on community cohesion and mental well-being (Finlay et al., 2012). A recent report on the displacement of First Nation communities in Canada documents the negative impacts that result from social isolation, lack of access to traditional food, job insecurity, lack of, or inconsistent access to health services, and poor psychological health outcomes (The Standing Senate Committee on Aboriginal Peoples, 2019). Moreover, previous research on health impacts of disasters on Indigenous communities has shown that social isolation and loss of a home and valuable possessions (including cultural possessions) can have considerable psychological and mental health impacts on Indigenous peoples and their family, and thus affecting their ability to cope during this difficult time (Montesanti et al., 2019). Furthermore, displacement and loss of land should be given significant consideration for Indigenous peoples following a disaster as land is an important determinant of Indigenous Peoples’ health (Reading & Wien, 2009). In our qualitative study, Elders, adults, and youth displayed and discussed a major sense of loss in relation to land. This is an important finding to consider as most non-Indigenous people view land as something they own or an asset whereas Indigenous peoples have a strong emotional and physical connection to land and nature. Prior research with Indigenous youth in Canada showed how connection to land, nature and spirituality helped youth cope with daily stressors and difficult situations (Hatala et al., 2020). Furthermore, Hatala et al. (2017) described how managing stress and promoting resilience in Indigenous youth is supported by nurturing a sense of belonging and fostering cultural continuity, which can include connection to land, spirituality, and traditions.

It is well known that inequities exist for health and mental health and access to services and supports for Indigenous peoples compared with non-Indigenous peoples (Pan-Canadian Health Inequalities Reporting Initiative, 2018). With heightened physical and emotional stress from a disaster, these inequities become even more visible. Our research highlighted not only the experiences of physical and emotional stress from the wildfire but also challenges that Indigenous residents faced when accessing services and supports for health and mental health. With limited support for emotional and physical disturbances following a traumatic event, the ability to cope is challenging. External support from local and provincial government agencies was underscored to be critical in assisting Indigenous communities to cope during and after the wildfire. Support from governments and agencies may enhance community and individual efficacy to take action and feel in control of their situation, promoting positive coping strategies (Brissette et al., 2020; Folkman & Moskowitz, 2000; Zakowski et al., 2001). However, participants described inadequate support for Indigenous residents, which was compounded by poor communication during the evacuation and recovery response. Concerns relating to support and communication are consistent with other research findings on the impacts of the Horse River wildfire in Indigenous communities (Willow Springs Strategic Solutions, 2018).

There is extensive research on positive coping strategies such as seeking support from others, staying connected to family and community and spirituality that can be protective factors against mental stress and promote well-being (Fleming & Ledogar, 2008; Folkman & Moskowitz, 2000). Research from Fleming and Ledogar (2008) found that incorporating traditional spirituality and cultural resources can promote coping and healing from trauma and unresolved historical grief for Indigenous peoples. In Indigenous contexts across Canada, the individual is understood as integrally woven into the collective fabric, based on kinship and social relationships. From a “collective coping” strategy, participants relayed how they supported their community members by delivering foods and supplies to Elders and residents and taking in evacuees. Gordon and colleagues found that residents with strong community connections showed better mental health 2 years post disaster (Gordon, 2011). Adding to this, we know that kinship relationships and social networks are known to contribute to Indigenous well-being and positive coping (Kirmayer et al., 2003; Kral, 2009; Kral & Schwab, 2003). Research links Indigenous resilience with cultural continuity, and community control and action (Allen et al., 2009; Chandler & Lalonde, 1998). The stories of Indigenous participants in the research reinforce understandings of community connectedness as a central concept in Indigenous coping and resilience in the context of a disaster. Moreover, a meaningful way to encourage connectedness among community members was through the therapeutic use of the sharing circle. Sharing circles are being used in a variety of settings as an alternative approach to manage stress and promote mental well-being (Mehl-Madrona & Mainguy, 2014).

## Research Implications

The results from this research highlight the importance of peer support, connection to land and culture, consistent and effective communication from governments and recognition of the transmission of historical loss that can be compounded by disasters. Unlike disaster-related evacuations in more accessible locations, such as urban areas or where people have the financial and other means to relocate themselves, residents in rural and remote Indigenous communities can be dependent on government resources or external assistance to evacuate. It was very evident that clear, conscience and consistent communication is imperative among governments and Indigenous leaders in communities from the start of the disaster through recovery. As we noted earlier, the findings of this research are relevant to the current COVID-19 pandemic context in Canada were Indigenous communities rely on strong government leadership and communication during pandemic response (Alberta, 2020).

Mental health practitioners can benefit from an understanding of coping resources and strategies among Indigenous peoples affected by a disaster to support recovery. For instance, culturally strengthening strategies for assisting with coping can include land-based healing activities. Furthermore, the therapeutic nature of the sharing circle could be utilized by mental health practitioners to allow residents to share their experience, thoughts, and feelings in a safe place.

The Truth and Reconciliation Commission of Canada concluded that greater value ought to be placed on Indigenous experience and to strengthen our relationships with Indigenous peoples of Canada. Part of this process of renewal depends on how the voices and experiences of Indigenous peoples and communities are heard and considered in the planning, mitigation, and recovery of future disasters and their impacts on Indigenous communities. This emphasizes the need for more CBPR and use of Indigenous research methodology to fully and appropriately understand Indigenous experiences. Today, news about COVID-19 in Canada and across the world is at the forefront of our minds. With this news, it is easy for us to feel stress, concern, and even fear. This global pandemic presents unique challenges for Indigenous communities, particularly Elders who are socially isolated and distancing from their kinship network. Our findings can be used to support positive coping and healing among Indigenous peoples in response to future public health emergencies (e.g., pandemics, flood, wildfire).

While our research findings offer important insights for future policy and practice, we would like to highlight a few limitations of our study. First, the rural sharing circle occurred 9 months after the three urban sharing circles were conducted and, therefore, the rural residents were in a different stage of recovery. Second, a purposive sampling strategy was used to recruit participants; thus, findings may not be generalizable to all Indigenous populations in the region.

## Conclusion

This research was unique in that it supported an intergenerational perspective and understanding of coping among diverse Indigenous residents following a disaster event. We applied CBPR approach, OCAP™ principles, and an Indigenous methodology to discern and develop a deeper understanding of what contributes to coping among Indigenous populations in the context of a disaster. An unexpected outcome of this research was the therapeutic value of the sharing circles for participants to share their experience. Sharing circles can be a powerful means of bringing some degree of healing to the mind, the heart, the body and the spirit. For many residents their participation in sharing circles was the first time they had openly spoke about their experiences with the wildfire with other Indigenous residents. Interestingly, adults and Elders both made a connection to historical loss and trauma and the current context of the disaster. The resilience developed to overcome long-term impacts of colonization and past trauma was used to recognize indigeneity as a strength which supported coping from the wildfire.

This research contributes to a better understanding of coping responses among Indigenous peoples and communities during and after a disaster event. We highlighted important considerations for governments, service providers, policy, and decision-makers to better support Indigenous peoples following a disaster or public health crisis. Relationship and connection are a cultural norm for many Indigenous peoples as well as an important requirement for the postdisaster health and well-being of First Nations and Métis residents in Wood Buffalo. Relationship to land, cultural belongings, spirituality, and social networks are important coping resources for Indigenous peoples and for healing following a disaster. Indigenous scholars have argued that colonization, historical oppression, and contemporary marginalization are all “disasters” for Indigenous communities (see, for example, Dunbar-Ortiz, 2014). Therefore, it is imperative to situate the experience of Indigenous peoples and their ability to cope with a disaster event within a sociohistorical colonial context.

## References

[bibr1-10497323211009194] Aldwin C. M. Sutton K. J. Lachman M. (1996). The development of coping resources in adulthood. Journal of Personality, 64(4), 837–871. 10.1111/j.1467-6494.1996.tb00946.x 8956515

[bibr2-10497323211009194] Aldwin C. M. (2007). Stress, coping, & development: An integrative perspective (2nd ed.). Guildford Press.

[bibr3-10497323211009194] Allen J. Mohatt G. Fok C. Henry D. , & Awakening Team. (2009). Suicide prevention as a community development process: Understanding circumpolar youth suicide prevention through community level outcomes. International Journal of Circumpolar Health, 68(3), 274–291. 10.3402/ijch.v68i3.18328 19705659 PMC2875412

[bibr4-10497323211009194] Archibald J. (2008). Indigenous storywork: Educating the heart, mind, body, and spirit. UBC Press.

[bibr5-10497323211009194] Asfaw H. W. McGee T. K. Christianson A. C. (2019). The role of social support and place attachment during hazard evacuation: The case of Sandy Lake First Nation, Canada. Environmental Hazards, 18(4), 361–381. 10.1080/17477891.2019.1608147

[bibr6-10497323211009194] Asfaw H. W. McGee T. K. Christianson A. C. (2020). Indigenous elders’ experiences, vulnerabilities and coping during hazard evacuation: The case of the 2011 Sandy Lake First Nation wildfire evacuation. Society & Natural Resources, 33, 1273–1291. 10.1080/08941920.2020.1745976

[bibr7-10497323211009194] Baciu A. Negussie Y. Geller A. Weinstein J. N. (2017). Communities in action: Pathways to health equity. 10.17226/24624 28418632

[bibr8-10497323211009194] Baum F. MacDougall C. Smith D. (2006). Participatory action research. Journal of Epidemiology and Community Health, 60(10), 854–857. 10.1136/jech.2004.028662 16973531 PMC2566051

[bibr9-10497323211009194] Beverly J. L. Bothwell P. (2011). Wildfire evacuations in Canada 1980–2007. Natural Hazards, 59(1), 571–596. 10.1007/s11069-011-9777-9

[bibr10-10497323211009194] Bihari M. Ryan R. (2012). Influence of social capital on community preparedness for wildfires. Landscape and Urban Planning, 106(3), 253–261. 10.1016/j.landurbplan.2012.03.011

[bibr11-10497323211009194] Birt L. Scott S. Cavers D. Campbell C. Walter F. (2016). Member checking: A tool to enhance trustworthiness or merely a nod to validation? Qualitative Health Research, 26(13), 1802–1811. 10.1177/1049732316654870 27340178

[bibr12-10497323211009194] Bohanon J. P. (2006). The Talking Circle: A perspective in culturally appropriate group work with Indigenous peoples [Paper presentation]. 6th International Conference on Diversity in Organizations, Communities & Nations, Southeastern Oklahoma State University, June, Durant, United States.

[bibr13-10497323211009194] Brissette C. N. Whyne Z. E. Lehrer H. M. Woo J. Steinhardt M. A. (2020). Associations of historical loss, resilience, and coping with loss-related emotional symptoms in the anishinaabe. American Journal of Health Behavior, 44(2), 244–251. 10.5993/ajhb.44.2.11 32019656

[bibr14-10497323211009194] Carr T. Sedgewick J. Roberts R. Groot G. (2020). The sharing circle method: Understanding Indigenous cancer stories. SAGE Research Methods Cases. https://methods.sagepub.com/case/the-sharing-circle-method-understanding-indigenous-cancer-stories

[bibr15-10497323211009194] Carroll M. Cohn P. Seesholtz D. Higgins L. (2005). Fire as a galvanizing and fragmenting influence on communities: The case of the Rodeo–Chediski fire. Society & Natural Resources, 18, 301–320. 10.1080/08941920590915224

[bibr16-10497323211009194] Chan J. To H.-P. Chan E. (2006). Reconsidering social cohesion: Developing a definition and analytical framework for empirical research. Social Indicators Research, 75(2), 273–302. 10.1007/s11205-005-2118-1

[bibr17-10497323211009194] Chandler M. Lalonde C. (1998). Cultural continuity as a hedge against suicide in Canada’s First Nations. Transcultural Psychiatry, 35(2), 191–219. 10.1177/136346159803500202

[bibr18-10497323211009194] Christianson A. (2015). Social science research on Indigenous wildfire management in the 21st century and future research needs. International Journal of Wildland Fire, 24(2), 190–200. 10.1071/wf13048

[bibr19-10497323211009194] Chun C. A. Moos R. H. Cronkite R. C. (2006). Culture: A fundamental context for the stress and coping paradigm. In Wong P. T. P. Wong L. C. J. (Eds.), Handbook of multicultural perspectives on coping (pp. 29–53). Springer.

[bibr20-10497323211009194] Constantine M. G. Alleyne V. L. Caldwell L. D. McRae M. B. Suzuki M. B. (2005). Coping responses of Asian, Black, and Latino/Latina New York City residents following the September 11, 2001 terrorist attacks against the United States. Cultural Diversity and Ethnic Minority Psychology, 11, 293–308. 10.1037/1099-9809.11.4.293 16478350

[bibr21-10497323211009194] Constantine M. G. Donnelly P. C. Myers L. J. (2002). Collective self-esteem and Africultural coping styles in African American adolescents. Journal of Black Studies, 32, 698–710. 10.1177/00234702032006004

[bibr22-10497323211009194] Dodd W. Scott P. Howard C. Scott C. Rose C. Cunsolo A. Orbinski J. (2018). Lived experience of a record wildfire season in the Northwest Territories, Canada. Canadian Journal of Public Health, 109(3), 327–337. 10.17269/s41997-018-0070-5 29981098 PMC6964492

[bibr23-10497323211009194] Dunbar-Ortiz R. (2014). An Indigenous peoples’ history of the United States (Vol. 3). Beacon Press.

[bibr24-10497323211009194] Enarson E. Chakrabarti P. D. (2009). Women, gender and disaster: Global issues and initiatives. SAGE.

[bibr25-10497323211009194] Finlay S. Moffat A. Gazzard R. Baker D. Murray V. (2012). Health impacts of wildfires. PLOS Currents Disasters. 10.1371/4f959951cce2c PMC3492003 23145351

[bibr26-10497323211009194] The First Nations Information Governance Centre. (2014). Ownership, Control, Access and Possession (OCAP^TM^): The path to first nations information governance. https://achh.ca/wp-content/uploads/2018/07/OCAP_FNIGC.pdf

[bibr27-10497323211009194] Fleming J. Ledogar R. J. (2008). Resilience, an evolving concept: A review of literature relevant to Aboriginal research. Pimatisiwin: A Journal of Aboriginal & Indigenous Community Health, 6(2), 7–23. PMC2956753 20963184

[bibr28-10497323211009194] Fletcher C. (2003). Community-based participatory research relationships with aboriginal communities in Canada: An overview of context and process. Pimatisiwin: A Journal of Aboriginal & Indigenous Community Health, 1(1), 27–62.

[bibr29-10497323211009194] Folkman S. (1984). Personal control and stress and coping processes: A theoretical analysis. Journal of Personality and Social Psychology, 46, 839–852. 10.1037/0022-3514.46.4.839 6737195

[bibr30-10497323211009194] Folkman S. Moskowitz J. T. (2000). Positive affect and the other side of coping. American Psychologist, 55(6), 647–654. 10.1037/0003-066x.55.6.647 10892207

[bibr31-10497323211009194] Gaylord-Harden N. K. Cunningham J. A. (2009). The impact of racial discrimination and coping strategies on internalizing symptoms in African American youth. Journal of Youth and Adolescence, 38, 532–543. 10.1007/s10964-008-9377-5 19636726

[bibr32-10497323211009194] Gbrich C. (2007). Qualitative data analysis: An introduction. SAGE.

[bibr33-10497323211009194] Ginige K. Amaratunga D. Haigh R. (2009). Mainstreaming gender in disaster reduction: Why and how? Disaster Prevention and Management: An International Journal, 18, 23–34. 10.1108/09653560910938510

[bibr34-10497323211009194] Gordon R. (2011, November). The course of recovery after disaster [Paper presentation]. CIMA Conference, Melbourne, Victoria, Australia.

[bibr35-10497323211009194] Hatala A. R. Njeze C. Morton D. Pearl T. Bird-Naytowhow K. (2020). Land and nature as sources of health and resilience among Indigenous youth in an urban Canadian context: A photovoice exploration. BMC Public Health, 20, Article 538. 10.1186/s12889-020-08647-z PMC7169029 32312240

[bibr36-10497323211009194] Hatala A. R. Pearl T. Bird-Naytowhow K. Judge A. Sjoblom E. Liebenberg L. (2017). “I have strong hopes for the future”: Time orientations and resilience among Canadian Indigenous youth. Qualitative Health Research, 27(9), 1330–1344. https://journals.sagepub.com/doi/10.1177/1049732317712489?url_ver=Z39.88-2003&rfr_id=ori:rid:crossref.org&rfr_dat=cr_pub%3dpubmed 28682711 10.1177/1049732317712489

[bibr37-10497323211009194] Heidi E. (2005). Reconsidering emergency management and Indigenous communities in Australia. Global Environmental Change Part B: Environmental Hazards, 6(1), 1–7. 10.1016/j.hazards.2004.08.001

[bibr38-10497323211009194] Hopkins P. E. (2007). Thinking critically and creatively about focus groups. Area, 39(4), 528–535. 10.1111/j.1475-4762.2007.00766.x

[bibr39-10497323211009194] Jogen C. McCalman J. Bainbridget R. (2020). A systematic scoping review of the resilience intervention literature for Indigenous adolescents in CANZUS nations. Frontiers in Public Health, 7, Article 351. 10.3389/fpubh.2019.00351 PMC6967740 31998674

[bibr40-10497323211009194] Joseph J. Kuo B. C. H. (2009). Black Canadians’ coping responses to racial discrimination. Journal of Black Psychology, 35, 78–101. 10.1177/0095798408323384

[bibr41-10497323211009194] Kading M. L. Gonzalez M. B. Herman K. A. Gonzalez J. Walls M. L. (2019). Living a good way of life: Perspectives from American Indian and First Nation young adults. American Journal of Community Psychology, 64(1–2), 21–33. 10.1002/ajcp.12372 31486101 PMC6800209

[bibr42-10497323211009194] Kendall E. Sunderland N. Barnett L. Nalder G. Matthews C. (2011). Beyond the rhetoric of participatory research in Indigenous communities: Advances in Australia over the last decade. Qualitative Health Research, 21(12), 1719–1728. 10.1177/1049732311418124 21844284

[bibr43-10497323211009194] Kim H. Sefcik J. S. Bradway C. (2017). Characteristics of qualitative descriptive studies: A systematic review. Research in Nursing & Health, 40(1), 23–42. 10.1002/nur.21768 27686751 PMC5225027

[bibr44-10497323211009194] Kirmayer L. Simpson C. Cargo M. (2003). Healing traditions: Culture, community and mental health promotion with Canadian Aboriginal peoples. Australasian Psychiatry, 11(Suppl. 1), S15–S23. 10.1046/j.1038-5282.2003.02010.x

[bibr45-10497323211009194] KPMG International. (2017). Regional municipality of Wood Buffalo: Lessons learned and recommendations from the 2016 Horse River wildfire. https://www.preventionweb.net/publications/view/54405

[bibr46-10497323211009194] Kral I. (2009). Oral to literate traditions: Emerging literacies in remote Aboriginal Australia. TESOL in Context, 19(2), 34–49.

[bibr47-10497323211009194] Kral I. Schwab R. (2003). The realities of Indigenous adult literacy acquisition and practice: Implications for capacity development in remote communities [Discussion paper]. The Australian National University.

[bibr48-10497323211009194] Lam A. G. Zane N. W. (2004). Ethnic differences in coping with interpersonal stressors: A test of self-construals as cultural mediators. Journal of Cross-Cultural Psychology, 35, 446–459. 10.1177/0022022104266108

[bibr49-10497323211009194] Langer E. L. McGee T. K. (2017). Wildfire risk awareness and prevention by predominantly Māori rural residents, Karikari Peninsula, Aotearoa New Zealand. International Journal of Wildland Fire, 26(9), 820–828. 10.1071/wf16133

[bibr50-10497323211009194] Latimer M. Sylliboy J. R. MacLeod E. Rudderham S. Francis J. Hutt-MacLeod D. Finley G. A. (2018). Creating a safe space for First Nations youth to share their pain. Pain Reports, 3, e682. 10.1097/pr9.0000000000000682 30324174 PMC6172818

[bibr51-10497323211009194] Lavallée L. F. (2009). Practical application of an Indigenous research framework and two qualitative Indigenous research methods: Sharing circles and Anishnaabe symbol-based reflection. International Journal of Qualitative Methods, 8(1), 21–40. 10.1177/160940690900800103

[bibr52-10497323211009194] Lawrence J. Ashford K. Dent P. (2006). Gender differences in coping strategies of undergraduate students and their impact on self-esteem and attainment. Active Learning in Higher Education, 7(3), 273–281. 10.1177/1469787406069058

[bibr53-10497323211009194] Lazarus R. S. Folkman S. (1984). Stress, appraisal, and coping. Springer.

[bibr54-10497323211009194] Loppie C. (2007). Learning from the grandmothers: Incorporating Indigenous principles into qualitative research. Qualitative Health Research, 17(2), 276–284. 10.1177/1049732306297905 17220397

[bibr55-10497323211009194] Matud M. P. (2004). Gender differences in stress and coping styles. Personality and Individual Differences, 37(7), 1401–1415. 10.1016/j.paid.2004.01.010

[bibr56-10497323211009194] McCampbell H. (2011). Sacred smoke: The ancient art of smudging for modern times. Native Voices books.

[bibr57-10497323211009194] McGee T. Nation M. Christianson A. (2019). Residents’ wildfire evacuation actions in Mishkeegogamang Ojibway Nation, Ontario, Canada. International Journal of Disaster Risk Reduction, 33, 266–274. 10.1016/j.ijdrr.2018.10.012

[bibr58-10497323211009194] Mehl-Madrona L. Mainguy B. (2014). Introducing healing circles and talking circles into primary care. The Permanente Journal, 18(2), 4–9. 10.7812/tpp/13-104 PMC4022550 24867544

[bibr59-10497323211009194] Mi’Kmaw spirituality—Talking circles. (2016). http://www.muiniskw.org/pgCulture2c.htm

[bibr60-10497323211009194] Montesanti S. Thurston W. Turner D. Medicine-Traveler R. (2019). A First Nations framework for emergency planning: A community-based response to the health and social effects from a flood. International Journal of Indigenous Health, 14(1), 85–106. 10.32799/ijih.v14i1.31952

[bibr61-10497323211009194] Neergaard M. A. Olesen F. Andersen R. S. Sondergaard J. (2009). Qualitative description—The poor cousin of health research? BMC Medical Research Methodology, 9(1), Article 52. 10.1186/1471-2288-9-52 PMC2717117 19607668

[bibr62-10497323211009194] Pan-Canadian Health Inequalities Reporting Initiative. (2018). Key health inequalities in Canada: A national portrait. https://www.canada.ca/content/dam/phac-aspc/documents/services/publications/science-research/key-health-inequalities-canada-national-portrait-executive-summary/hir-full-report-eng.pdf

[bibr63-10497323211009194] Pope C. Ziebland S. Mays N. (2006). Analysing qualitative data. In Pope C. Mays N. (Eds.), Qualitative research in health care (3rd ed., pp. 63–81). Blackwell Publishing.

[bibr64-10497323211009194] Reading C. L. Wien F. (2009). Health inequalities and social determinants of aboriginal peoples’ health. National Collaborating Centre for Indigenous Health.

[bibr65-10497323211009194] Renk K. Creasey G. (2003). The relationship of gender, gender identity, and coping strategies in late adolescents. Journal of Adolescence, 26(2), 159–168. 10.1016/s0140-1971(02)00135-5 12581724

[bibr66-10497323211009194] Rice P. L. Ezzy D. (1999). Qualitative research methods: A health focus. Oxford University Press.

[bibr67-10497323211009194] Rivera F. I. (2012). Cultural mechanisms in the exchange of social support among Puerto Ricans after a natural disaster. Qualitative Health Research, 22(6), 801–809. 10.1177/1049732311432719 22232298 PMC3343180

[bibr68-10497323211009194] Sandelowski M. (2000). Whatever happened to qualitative description? Research in Nursing & Health, 23(4), 334–340. 10.1002/1098-240x(200008)23:4<334::aid-nur9>3.0.co;2-g 10940958

[bibr69-10497323211009194] Sheu H. B. Sedlacek W. H. (2004). An exploratory study of help-seeking attitudes and coping strategies among college students by race and gender. Measurement and Evaluation in Counseling and Development, 37(3), 130–143. 10.1080/07481756.2004.11909755

[bibr70-10497323211009194] Spence P. R. Lachlan K. A. Burke J. M. (2007). Adjusting to uncertainty: Coping strategies among the displaced after Hurricane Katrina. Sociological Spectrum, 27(6), 653–678. 10.1080/02732170701533939

[bibr71-10497323211009194] Sullivan-Bolyai S. Bova C. Harper D. (2005). Developing and refining interventions in persons with health disparities: The use of qualitative description. Nursing Outlook, 53(3), 127–133. 10.1016/j.outlook.2005.03.005 15988449

[bibr72-10497323211009194] Tachine A. R. Bird E. Y. Cabrera N. L. (2016). Sharing circles: An Indigenous methodological approach for researching with groups of Indigenous peoples. International Review of Qualitative Research, 9(3), 277–295. 10.1525/irqr.2016.9.3.277

[bibr73-10497323211009194] Taylor S. E. Welch W. T. Kim H. S. Sherman D. K. (2007). Cultural differences in the impact of social support on psychological and biological stress responses. Psychological Science, 18, 831–837. 10.1111/j.1467-9280.2007.01987.x 17760781

[bibr74-10497323211009194] The Standing Senate Committee on Aboriginal Peoples. (2019). How did we get here? A concise, unvarnished account of the history of the relationship between Indigenous Peoples and Canada (pp. 1–58). Ontario.

[bibr75-10497323211009194] Thomas D. Mitchell T. Courtney A . (2015). Re-evaluating resilience: From individual vulnerabilities to strength of cultures and collectives among Indigenous communities. Resilience, 42(2), 116–129. 10.1080/21693293.2015.1094174

[bibr76-10497323211009194] Torsch V. L. Ma G. X. (2000). Cross-cultural comparison of health perceptions, concerns, and coping strategies among Asian and Pacific Islander American elders. Qualitative Health Research, 10(4), 471–489. 10.1177/104973200129118589 11010073

[bibr77-10497323211009194] Townshend I. Awosoga O. Kulig J. Fan H. (2015). Social cohesion and resilience across communities that have experienced a disaster. Natural Hazards, 76, 913–938. 10.1007/s11069-014-1526-4

[bibr78-10497323211009194] Whittemore R. Chase S. K. Mandle C. L. (2001). Validity in qualitative research. Qualitative Health Research, 11(4), 522–537. 10.1177/104973201129119299 11521609

[bibr79-10497323211009194] Willow Springs Strategic Solutions. (2018, October 31). Rebuilding resilient Indigenous communities—Report and video public release. http://www.willowspringsss.com/blog/rebuilding-resilient-indigenous-communities-report-and-video-public-release

[bibr80-10497323211009194] Wong P. T. P. Wong L. C. J. (2006). Handbook of multicultural perspectives on stress and coping. Springer.

[bibr81-10497323211009194] Zakowski S. G. Hall M. H. Klein L. C. Baum A. (2001). Appraised control, coping, and stress in a community sample: A test of the goodness-of-fit hypothesis. Annals of Behavioral Medicine, 23(3), 158–165. 10.1207/s15324796abm2303_3 11495216

